# Low Intrahepatic Distant Recurrence Rate Following RFA Using Linear Mode in Patients With Hepatocellular Carcinoma

**DOI:** 10.1002/jgh3.70145

**Published:** 2025-04-01

**Authors:** Hiroaki Takaya, Tadashi Namisaki, Yusuke Komeda, Hiroki Kinoshita, Naoki Nishimura, Yuki Tsuji, Shinya Sato, Norihisa Nishimura, Ko Saito, Shigeyuki Aizawa, Chie Morioka, Ryuichi Noguchi, Motoyuki Yoshida, Kosuke Kaji, Hitoshi Yoshiji

**Affiliations:** ^1^ Department of Gastroenterology Nara Prefecture Seiwa Medical Center Nara Japan; ^2^ Department of Gastroenterology Nara Medical University Nara Japan

**Keywords:** hepatocellular carcinoma, radiofrequency ablation, recurrence rate

## Abstract

**Background:**

Hepatocellular carcinoma (HCC) is one of the most common cancers worldwide. Radiofrequency ablation (RFA) can be utilized in elderly patients and those with cirrhosis with reduced functional liver reserve as it is less invasive. The arfa RFA system is the first system to offer a linear mode. However, the differences in performance between the linear and existing (nonlinear) modes remain unknown.

**Aim:**

This retrospective observational study compared the performance of the linear (linear group) and nonlinear RFA modes (nonlinear group) in HCC.

**Methods:**

Data of 425 patients with one to three HCC tumors measuring ≤ 3 cm who underwent RFA were analyzed. Recurrence (local and distant), survival, and complication rates between the linear and nonlinear groups were determined.

**Results:**

The intrahepatic distant recurrence rate was lower in the linear group than in the nonlinear group (*p* < 0.05). Multivariate analysis showed that the high platelet count, low AFP‐L3 levels, initial case, and linear mode were independent factors associated with a low intrahepatic distant recurrence rate following RFA. Liver disease‐related survival, HCC survival, overall survival of the initial HCC, local recurrence, and complication rates were comparable between the linear and nonlinear groups.

**Conclusion:**

The linear mode of the RFA protocol results in a lower intrahepatic distant recurrence rate compared with the nonlinear protocol.

## 1 Introduction

Primary liver cancer is the seventh most common cancer worldwide and the second leading cause of cancer death [1], with hepatocellular carcinoma (HCC) accounting for approximately 75% of cases [1, 2]. In Japan, HCC management follows consensus‐based clinical practice guidelines of the Japan Society of Hepatology (JSH) [3]. For patients with one to three HCC tumors measuring ≤ 3 cm, radiofrequency ablation (RFA) or resection is recommended [3]. Notably, the prognosis of patients with HCC treated with RFA and those who underwent resection is not significantly different [4, 5, 6]. Due to the increasing number of elderly patients with hepatitis C, which is a primary cause of liver cirrhosis [7], the prevalence of elderly patients with HCC in Japan is also increasing [8, 9]. Compared with resection, RFA is less invasive and can be utilized in elderly patients with reduced performance status and those with liver cirrhosis who have deteriorated functional liver reserve.

The RFA protocol usually employs a low‐power technique with manual multistep power increments to prevent popping, which results from a rapid increase in tumor temperature [10]. Popping is thought to result in tumor dissemination [11, 12]. The arfa RFA system (Japan Lifeline Co. Ltd., Tokyo, Japan) is the first system to offer a linear mode that automatically increases power at a rate of 1 W/6 s until the so‐called “roll‐off” or “break” (i.e., discontinuation of the delivery of the RFA system) is reached. We reported that the ablation area using the linear mode of the RFA protocol was larger than that of the existing RFA protocol [13]. However, the difference in recurrence and survival rates between the linear mode and existing RFA modes remains unexplored.

In this study, we determined whether the linear mode of the RFA protocol prevents recurrence and increases survival rates compared with the existing RFA protocol.

## 2 Materials and Methods

### 2.1 Patients and Study Design

**FIGURE 1:**
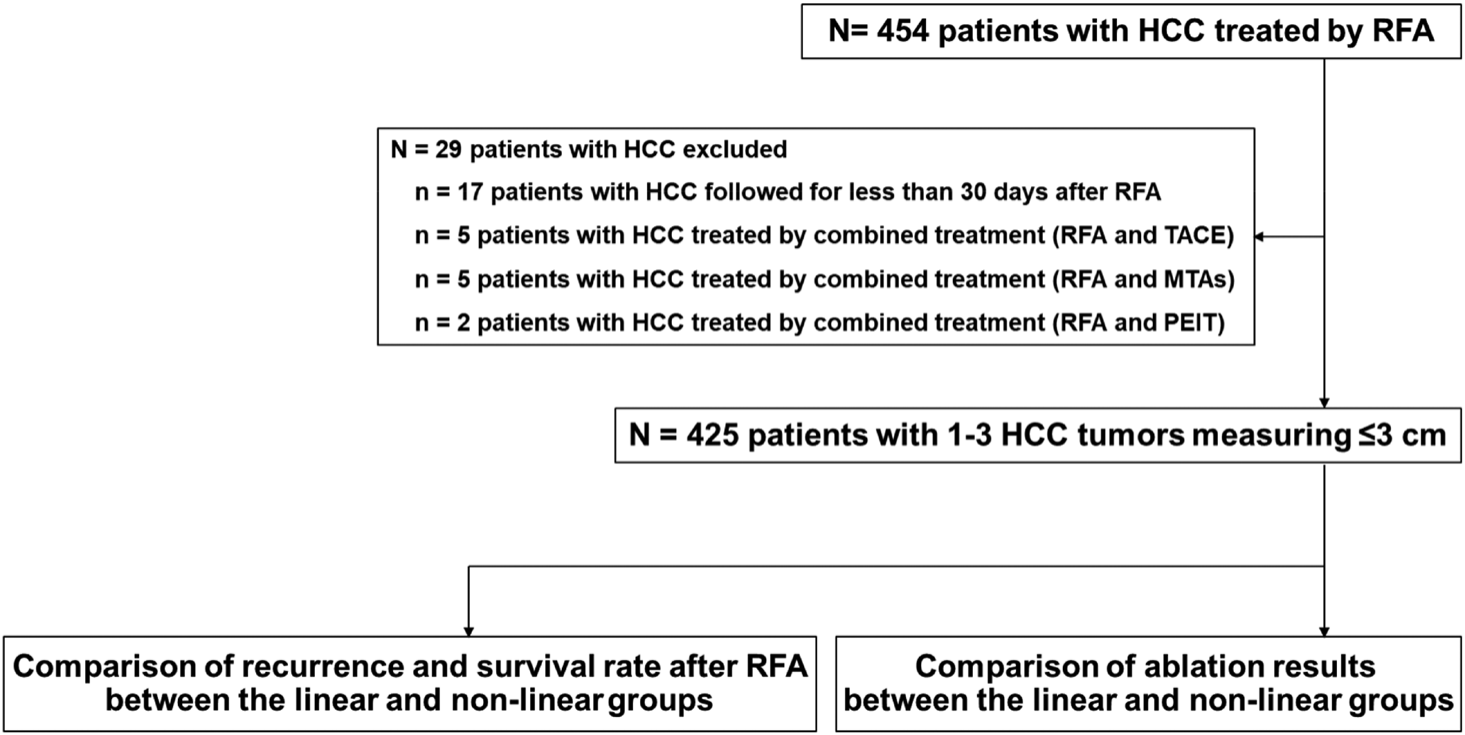
Study design. We first evaluated the recurrence and survival rates in patients with HCC following linear and nonlinear RFA. Next, we evaluated the ablation results in both groups. HCC, hepatocellular carcinoma; MTA, molecular target agent; PEIT, percutaneous ethanol injection; RFA, radiofrequency ablation; TACE, transcatheter arterial chemoembolization.

This retrospective observational study enrolled 454 patients with HCC who underwent RFA at Nara Medical University and Nara Prefecture Seiwa Medical Center between April 2016 and October 2023. HCC was confirmed based on dynamic computed tomography (CT) and/or dynamic magnetic resonance imaging (MRI) according to the JSH consensus‐based clinical practice guidelines for HCC management [3]. The inclusion criterion was patients with 1–3 HCC tumors that measured ≤ 3 cm. Meanwhile, patients with HCC who were treated with combination treatments (RFA and transcatheter arterial chemoembolization [14, 15], molecular targeted agents, or percutaneous ethanol injection) were excluded. Additionally, patients with HCC who could not be followed for > 30 days following RFA were excluded. Overall, 425 patients with HCC classified as Child–Pugh class A or B and without extrahepatic metastasis or vascular invasion were included in the final analysis [16]. Of these, 177 and 248 patients underwent RFA using the linear (linear group) and existing (nonlinear group) RFA protocols. Recurrence and survival rates following RFA as well as ablation results were analyzed in both patient groups (Figure 1). This study was approved by the local ethics committee of Nara Medical University and Nara Prefecture Seiwa Medical Center and was conducted in accordance with the ethical standards of the Declaration of Helsinki. Informed consent was obtained from all patients on an opt‐out basis.

### 2.2 RFA Treatment

RFA was performed using the arfa and VIVA RFA systems with adjustable electrode needles. The VIVA system was utilized from April 2016 to September 2020, while the arfa RFA system was utilized from October 2020 to December 2023. All RFA treatments were conducted percutaneously under ultrasound guidance (LOGIQ E9 XDclear 2.0, GE Healthcare or LOGIQ E10x, GE Healthcare) with intravenous conscious sedation and vital signs monitoring. RFA protocols were initiated at 40 W (2 cm 17G adjustable electrode needle), 50 W (2.5 cm 17G adjustable electrode needle), or 60 W (3 cm 17G adjustable electrode needle). The power of arfa was increased at a rate of 1 W/6 s (linear mode) until the so‐called “roll‐off” or “break” was reached, indicating discontinuation of the RFA system delivery. Similarly, the power of VIVA was increased at a rate of 10 W/min (nonlinear mode) until the so‐called “roll‐off” or “break” was reached. This protocol was repeated three times per RFA session.

### 2.3 Follow‐Up

All patients underwent blood examinations and dynamic CT or dynamic MRI every 1–4 months following RFA.

### 2.4 Statistical Analysis

Statistical analyses were conducted using EZR (version 1.63, Saitama Medical Center, Jichi Medical University), which is a graphical user interface for R (version 4.3.1, R Foundation for Statistical Computing, https://www.r‐project.org). EZR is a modified version of R commander version 2.80 that includes statistical functions frequently used in biostatistics [17]. Results were reported as median and interquartile range. Differences between the groups were analyzed using the Mann–Whitney *U* test, and Fisher's exact test was used to analyze categorical data. Recurrence and survival rates following RFA in both groups were analyzed using Gray's model and the log‐rank test. To determine the risk factors of intrahepatic distant recurrence following RFA, univariate and multivariate analyses using the Cox proportional hazard model were performed. A *p*‐value of < 0.05 (two‐tailed) was considered statistically significant.

## 3 Results

### 3.1 Characteristics of Patients With HCC in Both Groups

Table 1 presents the characteristics of the patients. The median age of the patients was 76 (69–81) years, and there were 296 men and 129 women. Overall, 79 patients had hepatitis B, 164 had hepatitis C, 67 had alcohol abuse, 85 had metabolic dysfunction‐associated steatohepatitis, 11 had autoimmune hepatitis, 5 had primary biliary cholangitis, and 14 had other diseases. The etiology of HCC was different between the linear and nonlinear groups (*p* < 0.05). Meanwhile, prothrombin times and platelet count were higher in the linear group than in the nonlinear group (both, *p* < 0.05). Additionally, the proportion of patients classified as Child–Pugh class A was higher in the linear group than in the nonlinear group (*p* < 0.05).

**TABLE 1:** Characteristics of patients in the linear and nonlinear groups.

Variable	Total (*n* = 425)	Linear group (*n* = 177)	Nonlinear group (*n* = 248)	*p* *
Age (years)	76 (69–81)	77 (71–82)	75 (68–79)	< 0.05
Sex (male/female)	296/129	121/56	175/73	NS
Etiology (HBV/HCV/alcohol abuse/MASH/AIH/PBC/others)	79/164/67/85/11/5/14	21/59/31/55/3/2/6	58/105/36/30/8/3/8	< 0.05
Albumin level (g/dL)	3.9 (3.6–4.2)	3.9 (3.6–4.3)	3.9 (3.6–4.2)	NS
Aspartate aminotransferase level (U/L)	30 (22–43)	29 (22–40)	31 (22–44)	NS
Alanine aminotransferase level (U/L)	22 (15–32)	21 (14–33)	23 (15–32)	NS
Total bilirubin level (mg/dL)	0.9 (0.7–1.2)	0.8 (0.7–1.3)	0.9 (0.7–1.2)	NS
Prothrombin time (%)	86 (75–96)	92 (82–102)	82 (72–89)	< 0.05
Platelet count (×10^4^/μL)	13.6 (9.9–17.6)	14.9 (10.8–18.7)	12.8 (9.6–17.1)	< 0.05
Child–Pugh score (5/6/7/8)	300/95/21/9	135/35/5/2	165/60/16/7	NS
Child–Pugh Class A/B	396/29	171/6	225/23	< 0.05
AFP (ng/mL)	4.8 (2.9–13.0)	4.5 (2.9–13.9)	5.2 (2.6–12.6)	NS
DCP (mAU/mL)	27 (16–58)	26 (16–49)	28 (16–64)	NS
AFP‐L3 (%)	0.5 (0.5–7.1)	0.5 (0.5–5.6)	0.5 (0.5–7.8)	NS
Initial/recurrence	126/299	45/132	81/167	NS
Maximum tumor size (cm)	1.7 (1.4–2.2)	1.7 (1.4–2.0)	1.8 (1.4–2.3)	NS
Tumor number (1/2/3)	353/64/8	154/22/1	199/42/7	NS

*Note:* Data are expressed as median (interquartile range). *p*‐Values represent comparisons between the linear group and the nonlinear group.

Abbreviations: AFP, alpha fetoprotein; AFP‐L3, lens culinaris agglutinin‐reactive alpha‐fetoprotein; AIH, autoimmune hepatitis; DCP, des‐γ‐carboxy prothrombin; HBV, hepatitis B virus; HCV, hepatitis C virus; MASH, metabolic dysfunction‐associated steatohepatitis; NS, not significant; PBC, primary biliary cholangitis.

* Linear group vs. nonlinear group.

### 3.2 Recurrence and Survival Rates After RFA in Both Groups

**FIGURE 2:**
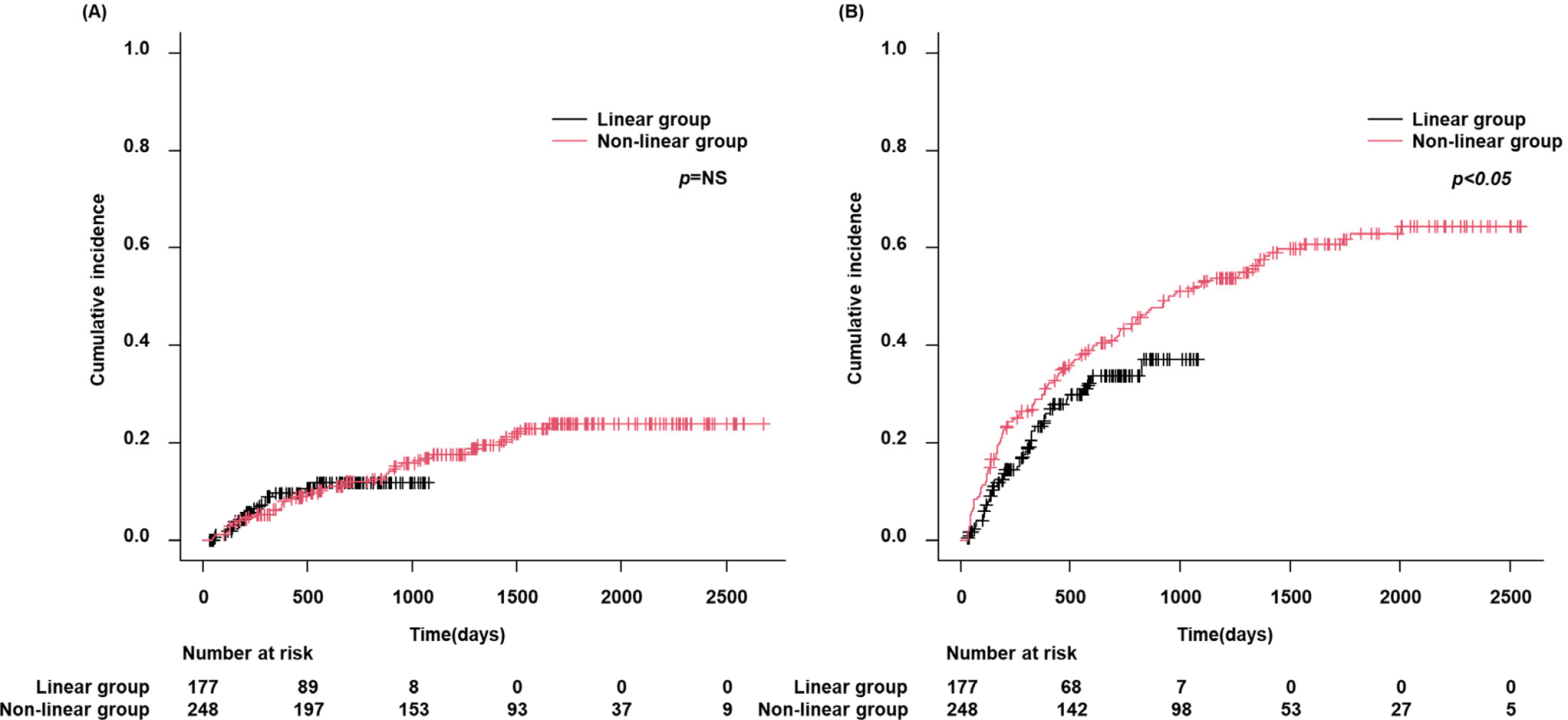
Recurrence rate following RFA in the linear and nonlinear groups. (A) Local recurrence rates were not different between the linear and nonlinear groups; (B) intrahepatic distant recurrence rates were lower in the linear group than in the nonlinear group (*p* < 0.05). RFA, radiofrequency ablation.

**FIGURE 3:**
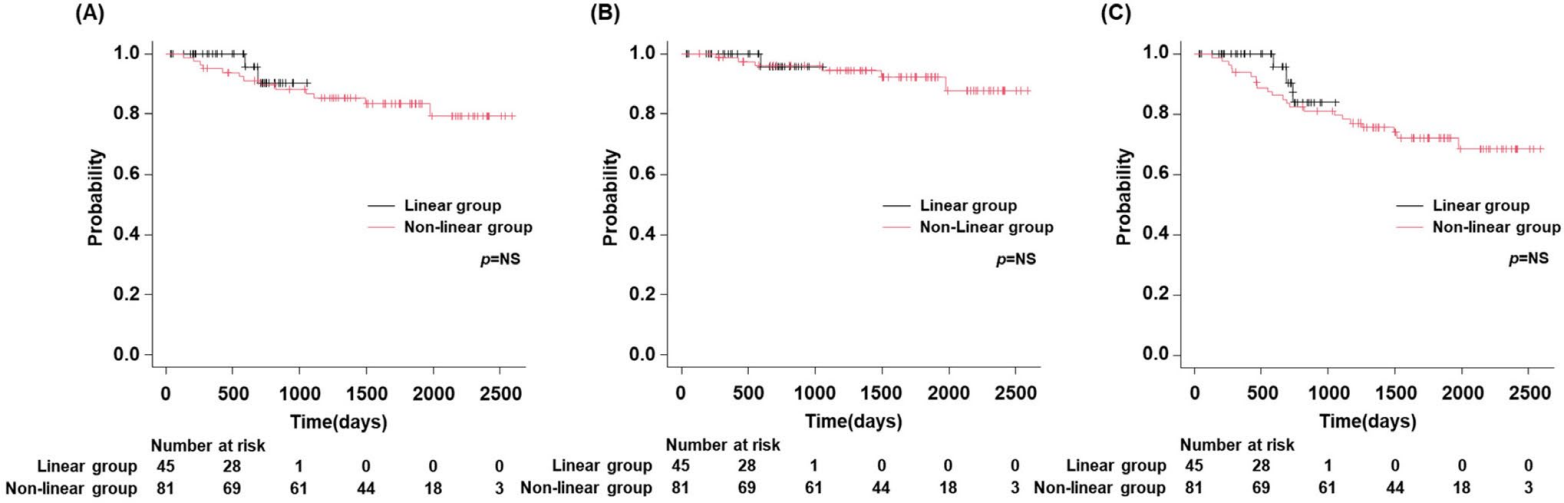
Survival rate following RFA in the linear and nonlinear groups. (A) Liver disease‐related survival; (B) HCC survival; (C) overall survival rates of initial HCC were not different between the linear and nonlinear groups. HCC, hepatocellular carcinoma; RFA, radiofrequency ablation.

**FIGURE 4:**
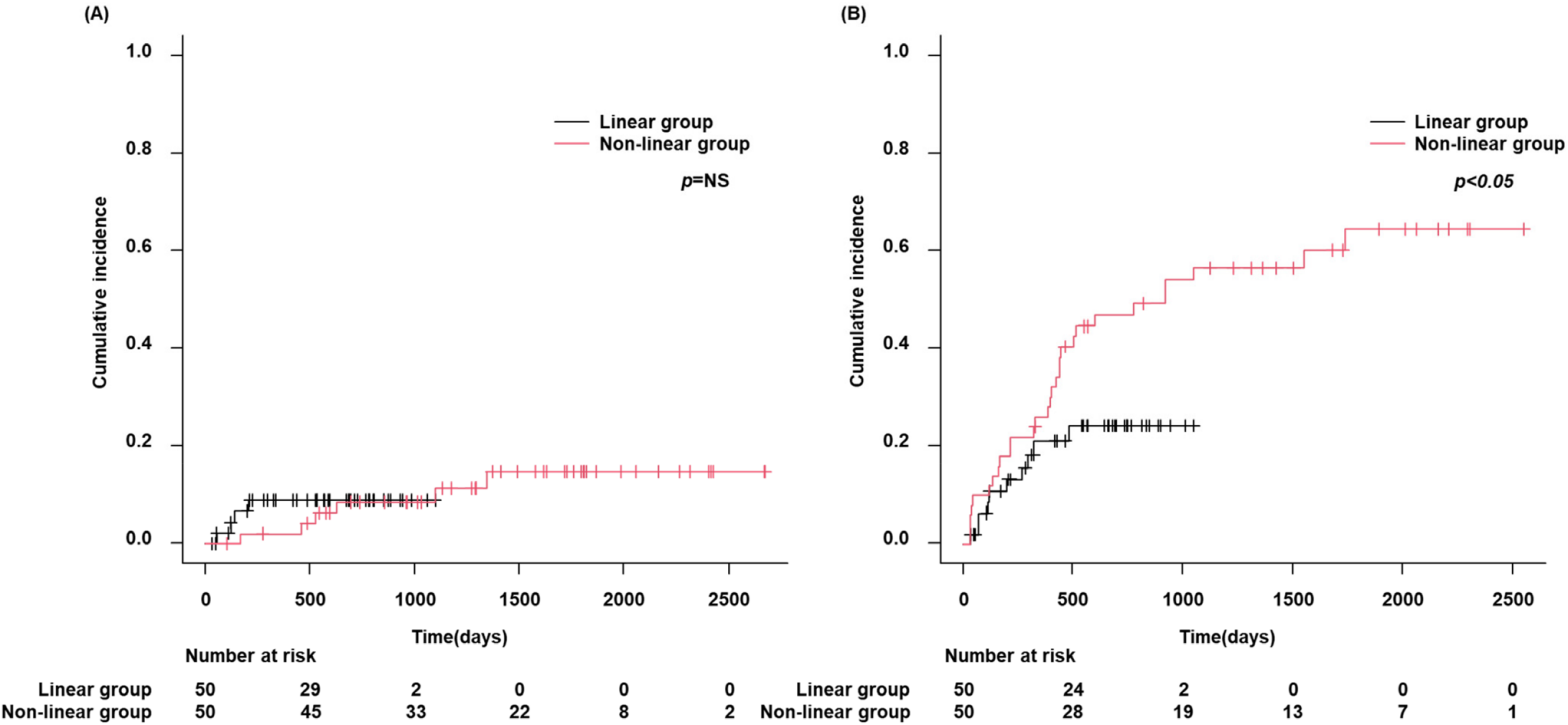
Recurrence rate following RFA in the linear and nonlinear groups after PSM. (A) Local recurrence rates were not different between the linear and nonlinear groups after PSM; (B) the intrahepatic distant recurrence rate was lower in the linear group than in the nonlinear group after PSM (*p* < 0.05). PSM, propensity score matching; RFA, radiofrequency ablation.

Although local recurrence rates after RFA were not significantly different between the linear and nonlinear groups (Figure 2A), the intrahepatic distant recurrence rate was lower in the linear group (*p* < 0.05) (Figure 2B). There were no significant differences in liver disease‐related survival, HCC survival, and overall survival rates between the linear and nonlinear groups for initial HCC (Figures 3A–C). Propensity score matching (PSM) was conducted using a logistic regression model that was adjusted for age, etiology, platelet count, and Child–Pugh class (Table 2). A greedy matching technique was applied to achieve a 1:1 ratio with a caliper width of 0.2 without replacement. After PSM, we analyzed the incidence of local and intrahepatic distant recurrence following RFA in both groups, revealing that although the local recurrence rate was not significantly different between the two groups (Figure 4A), the linear group had a lower rate of intrahepatic distant recurrence than the nonlinear group (*p* < 0.05) (Figure 4B).

**TABLE 2:** Characteristics of patients in the linear and nonlinear groups after propensity score matching.

Variable	Total (*n* = 100)	Linear group (*n* = 50)	Nonlinear group (*n* = 50)	*p* *
Age (years)	78 (73–82)	78 (73–82)	78 (73–82)	NS
Sex (male/female)	65/35	32/18	33/17	NS
Etiology (HBV/HCV/alcohol abuse/MASH/AIH/PBC/others)	14/48/20/18/0/0/0	7/24/10/9/0/0/0	7/24/10/9/0/0/0	NS
Albumin level (g/dL)	4.0 (3.8–4.2)	4.0 (3.8–4.2)	4.0 (3.8–4.3)	NS
Aspartate aminotransferase level (U/L)	28 (22–36)	28 (23–35)	26 (21–36)	NS
Alanine aminotransferase level (U/L)	18 (13–28)	18 (16–26)	18 (13–31)	NS
Total bilirubin level (mg/dL)	0.8 (0.6–1.2)	0.8 (0.7–1.3)	0.8 (0.6–1.1)	NS
Prothrombin time (%)	90 (80–97)	94 (86–98)	84 (76–93)	< 0.05
Platelet count (×10^4^/μL)	13.6 (11.5–17.1)	13.6 (11.3–17.0)	13.7 (11.5–17.1)	NS
Child–Pugh score (5/6/7/8)	78/20/2/0	41/8/1/0	37/12/1/0	NS
Child–Pugh class A/B	98/2	49/1	49/1	NS
AFP (ng/mL)	4.6 (3.0–12.9)	4.4 (3.1–13.0)	5.0 (2.9–12.7)	NS
DCP (mAU/mL)	26 (16–55)	23 (15–47)	27 (17–74)	NS
AFP‐L3 (%)	0.5 (0.5–8.7)	0.5 (0.5–1.0)	0.5 (0.5–16.5)	NS
Initial/recurrence	23/77	10/40	13/37	NS
Maximum tumor size (cm)	1.7 (1.4–2.1)	1.7 (1.2–2.0)	1.6 (1.2–2.4)	NS
Tumor number (1/2/3)	86/13/1	45/5/0	41/8/1	NS

*Note:* Data are expressed as median (interquartile range). *p*‐Values represent comparisons between the linear group and the nonlinear group.

Abbreviations: AFP, alpha fetoprotein; AFP‐L3, lens culinaris agglutinin‐reactive alpha‐fetoprotein; AIH, autoimmune hepatitis; DCP, des‐γ‐carboxy prothrombin; HBV, hepatitis B virus; HCV, hepatitis C virus; MASH, metabolic dysfunction‐associated steatohepatitis; NS, not significant; PBC, primary biliary cholangitis.

* Linear group vs. nonlinear group.

### 3.3 Ablation Results for Tumors in the Linear and Nonlinear Groups

The number of punctures and ablation times for 505 tumors in 425 patients was then assessed. Patients in the linear group experienced fewer punctures for each tumor compared with those in the nonlinear group (*p* < 0.05) (Table 3). Additionally, ablation times for each tumor were longer in the linear group than in the nonlinear group (*p* < 0.05) (Table 3).

**TABLE 3:** Ablation results for each tumor in the linear and nonlinear groups.

	Linear group	Nonlinear group	*p* *
Single puncture/two punctures/three or more punctures	172/28/1	213/86/5	< 0.05
Ablation time (s)	480 (380–626)	390 (280–535)	< 0.05

*Note:* Data are expressed as median (interquartile range). *p*‐Values represent comparisons between the linear group and the nonlinear group.

* Linear group vs. nonlinear group.

### 3.4 Ablation Results in Patients in the Linear and Nonlinear Groups

The total number of punctures in each patient was lower in the linear group than in the nonlinear group (*p* < 0.05) (Table 4). Additionally, popping was evaluated in 410 patients, revealing that the rate of popping was lower in the linear group than in the nonlinear group (*p* < 0.05) (Table 4). Meanwhile, complication rates were not significantly different between the two groups (Table 4). In the linear group, there were two cases of portal vein thrombosis, one case of intraabdominal hemorrhage, one case of intestinal perforation, and one case of hepatic infarction after RFA. In the nonlinear group, there was one case of cardiac tamponade, one case of portal vein thrombosis, one case of intraabdominal hemorrhage, and one case of hepatic infarction after RFA.

**TABLE 4:** Ablation results for each patient in the linear and nonlinear groups.

	Linear group	Nonlinear group	*p* *
Single puncture/two punctures/three or more punctures	131/38/8	130/96/22	< 0.05
Popping (with/without)	26/147	56/181	< 0.05
Complications (with/without)	5/172	4/244	NS

*Note:* Among patients in the linear group with a single tumor, 131 had a single puncture, 22 had two punctures, and 1 had three punctures. Among patients in the linear group with two tumors, 16 had two punctures, and 6 had three or more punctures. Among patients in the linear group with three tumors, 1 had three or more punctures. Among patients in the nonlinear group with a single tumor, 130 had single puncture, 68 had two punctures, and 1 had three punctures. Among patients in the nonlinear group with two tumors, 28 had two punctures, and 14 had three or more punctures. Among patients in the nonlinear group with three tumors, 7 had three or more punctures. Popping was evaluated in 410 patients. Data are expressed as median (interquartile range). *p*‐values represent comparisons between the linear group and the nonlinear group.

Abbreviation: NS, not significant.

* Linear group vs. nonlinear group.

### 3.5 Association Between Intrahepatic Distant Recurrence Rate and Patient or RFA System Characteristics

Patients were subsequently categorized into two groups on the basis of the existence of intrahepatic distant recurrence following RFA (Table 5). Patients with intrahepatic distant recurrence following RFA were younger than those without (*p* < 0.05). Patients with intrahepatic distant recurrence following RFA exhibited higher total bilirubin and lens culinaris agglutinin‐reactive alpha fetoprotein (AFP‐L3) levels than those without intrahepatic distant recurrence following RFA (both, *p* < 0.05). In addition, patients with intrahepatic distant recurrence after RFA had lower prothrombin times and platelet counts than those without intrahepatic distant recurrence after RFA (both, *p* < 0.05). Moreover, patients with intrahepatic distant recurrence following RFA had a higher proportion of cases classified as Child–Pugh class B, recurrence, and nonlinear mode of RFA protocol than those without intrahepatic distant recurrence following RFA (all, *p* < 0.05). Patients with intrahepatic distant recurrence following RFA had a higher total number of punctures than those without intrahepatic distant recurrence following RFA (*p* < 0.05). Univariate analysis demonstrated that platelet count, AFP‐L3 level, initial/recurrence, puncture number for each patient, and linear/nonlinear mode were associated with intrahepatic distant recurrence following RFA (Table 6). To identify factors independently associated with intrahepatic distant recurrence following RFA, multivariate analysis was performed using platelet count, Child–Pugh class, AFP‐L3 level, initial/recurrence, puncture number for each patient, and linear/nonlinear mode that had *p* < 0.1 in the univariate analysis. Nonmulticollinearity was confirmed using the variance inflation factor [18] between the linear/nonlinear mode and other factors, whose value was approximately 1.0 [19]. In the multivariate analysis, high platelet count, low AFP‐L3 level, initial case, and linear mode were significantly associated with a lower intrahepatic distant recurrence following RFA (Table 6).

**TABLE 5:** Characteristics of patients with or without intrahepatic distant recurrence after RFA.

Variable	Without intrahepatic distant recurrence (*n* = 243)	With intrahepatic distant recurrence (*n* = 182)	*p* *
Age (years)	76 (70–82)	75 (68–80)	< 0.05
Sex (male/female)	163/80	133/49	NS
Etiology (HBV/HCV/alcohol abuse/MASH/AIH/PBC/others)	46/80/42/53/8/4/10	33/84/25/32/3/1/4	NS
Albumin level (g/dL)	3.9 (3.6–4.2)	4.0 (3.7–4.2)	NS
Aspartate aminotransferase level (U/L)	30 (22–43)	29 (22–39)	NS
Alanine aminotransferase level (U/L)	21 (14–32)	23 (16–32)	NS
Total bilirubin level (mg/dL)	0.8 (0.6–1.1)	1.0 (0.7–1.4)	< 0.05
Prothrombin time (%)	89 (78–100)	82 (73–90)	< 0.05
Platelet count (×10^4^/μL)	15.0 (10.8–18.5)	12.5 (9.2–16.3)	< 0.05
Child–Pugh score (5/6/7/8)	183/48/7/5	117/47/14/4	< 0.05
Child–Pugh Class A/B	232/11	164/18	< 0.05
AFP (ng/mL)	4.7 (2.8–11.7)	6.5 (3.4–23.1)	NS
DCP (mAU/mL)	26 (16–58)	32 (17–54)	NS
AFP‐L3 (%)	0.5 (0.5–6.8)	1.5 (0.5–7.8)	< 0.05
Initial/recurrence	85/158	41/141	< 0.05
Maximum tumor size (cm)	1.7 (1.4–2.1)	1.7 (1.4–2.2)	NS
Tumor number (1/2/3)	206/34/3	147/30/5	NS
Ablation mode (linear/nonlinear)	132/111	45/137	< 0.05
Puncture number in each patient (1/2/3 or more)	156/77/10	105/57/20	< 0.05
Popping occurrence rate (%) (with/without)	18.1% (43/195)	22.7% (39/133)	NS

*Note:* Data are expressed as medians (interquartile ranges). *p*‐values represent comparisons between patients with intrahepatic distant recurrence and those without it. Popping occurrence is evaluated in 410 patients.

Abbreviations: AFP, alpha fetoprotein; AFP‐L3, lens culinaris agglutinin‐reactive alpha fetoprotein; AIH, autoimmune hepatitis; DCP, des‐γ‐carboxy prothrombin; HBV, hepatitis B virus; HCV, hepatitis C virus; MASH, metabolic dysfunction‐associated steatohepatitis; NS, not significant; PBC, primary biliary cholangitis.

* Patients with intrahepatic distant recurrence vs. those without intrahepatic distant recurrence.

**TABLE 6:** The association of patient characteristics and intrahepatic distant recurrence after RFA.

Variable	Univariate analysis			Multivariate analysis		
	HR	95% CI	*p*	HR	95% CI	*p*
Age ≥ 75 years old	0.99	0.742–1.334	0.9728			
Platelet count > 15 × 10^4^/μL	0.64	0.474–0.872	0.0045	0.68	0.486–0.940	0.0201
Child–Pugh Class A vs. B	0.63	0.385–1.019	0.0598	0.81	0.481–1.374	0.4398
AFP‐L3 < 10%	0.61	0.428–0.864	0.0055	0.64	0.441–0.921	0.0164
Initial vs. recurrence	0.54	0.378–0.760	0.0005	0.54	0.373–0.782	0.0011
Puncture number for each patient ≤ 2	0.55	0.346–0.878	0.0122	0.63	0.386–1.023	0.0618
Linear mode vs. nonlinear mode	0.69	0.485–0.976	0.0013	0.67	0.464–0.974	0.0360

Abbreviations: AFP‐L3, lens culinaris agglutinin‐reactive alpha‐fetoprotein; CI, confidence interval; HR, hazard ratio.

## 4 Discussion

This study revealed that the intrahepatic distant recurrence rate was lower in patients who underwent RFA using the linear mode when compared to those who underwent RFA using the existing protocol. Regarding popping, it was suggested that it is better controlled when the power is increased slowly over time [12]. Our results revealed that the rate of popping occurrence was lower in the linear group than in the nonlinear group, but it was not associated with the risk of intrahepatic distant recurrence after RFA [20]. Low platelet count was associated with intrahepatic distant recurrence following RFA in this study and in previous studies [21]. This study showed that platelet count was higher in the linear group than in the nonlinear group. However, the linear group had a lower rate of intrahepatic distant recurrence than the nonlinear group, even after PSM that adjusted for platelet count. AFP and des‐γ‐carboxy prothrombin (DCP) levels are reportedly associated with intrahepatic distant recurrence following RFA [22, 23]. Although patients with intrahepatic distant recurrence following RFA demonstrated higher AFP‐L3 levels than those without intrahepatic distant recurrence following RFA, this study showed that AFP, DCP, and AFP‐L3 levels were not significantly different between the linear and nonlinear groups. Another study reported that needle liver biopsy causes hematogenous dissemination of HCC [24]. In this study, the number of punctures during RFA was higher in the nonlinear group than in the linear group. Additionally, patients with intrahepatic distant recurrence after RFA had a higher number of punctures than those without intrahepatic distant recurrence. However, the number of punctures during RFA was not associated with the risk of intrahepatic distant recurrence after RFA.

Previous studies have reported that several cases of metastatic lung cancer spontaneously regress following RFA or microwave ablation (MWA) of a solitary metastatic lesion [25, 26]. A previous study indicated that distant tumors in a murine HCC model following RFA had a higher number of infiltrating CD8^+^ T lymphocytes than those before RFA [27]. Furthermore, MWA enhances tumor‐specific immune responses, including interleukin‐5, interleukin‐10, and interferon‐y, in patients with HCC [28]. Another study reported that the tumor necrosis factor‐α levels of the murine HCC model following MWA were higher in the high‐power MWA group than in the low‐power MWA group, and the high‐power MWA group showed a higher number of infiltrating CD8^+^ T lymphocytes in distant tumors than the low‐power MWA group [29]. The ablation may induce the abscopal effect, which is stronger in high‐power ablation than in low‐power ablation. The linear group exhibited a longer ablation time than the nonlinear group. Consequently, the linear group had a higher total amount of energy than the nonlinear group. Moreover, owing to the stronger abscopal effect induced in the linear group, it exhibited a lower intrahepatic distant recurrence rate than the nonlinear group.

This study revealed that the number of punctures for each tumor was lower in the linear group than in the nonlinear group. As slowly increasing power over time resulted in a wide ablation area [30], our findings suggest that the linear mode of the RFA protocol can ablate a larger area [13] and may reduce the number of punctures required. Therefore, the total number of punctures in each patient was lower in the linear group than in the nonlinear group. This study also revealed that the local recurrence rates were not significantly different between the linear and nonlinear groups. As the ablation area is usually monitored during RFA, a larger ablation area or fewer punctures may not have been associated with local recurrence. However, for inexperienced hepatologists, fewer punctures in RFA offer a significant advantage.

The survival rate was not significantly different between the linear and the nonlinear groups. However, the intrahepatic distant recurrence rate was lower in the linear group than in the nonlinear group in the present study. A previous study reported no difference in the survival rates between the patients with initial HCC and recurrent HCC after RFA [31]. Additionally, there was no difference in survival rates between patients with local and intrahepatic distant recurrence following RFA [31]. Repeated RFA using linear mode may be effective for intrahepatic recurrent HCC, and reducing the recurrence rate can lower hospitalization rates, resulting in reduced healthcare costs and less invasiveness for patients undergoing repeat RFA.

This study had some limitations, including its two‐center study design and small sample size. We used the arfa and VIVA RFA systems for comparing the performance between the linear and nonlinear modes of the RFA protocol. However, these systems employ a 17‐G adjustable electrode needle and are connected to a 200‐W generator [12] and are used as instruments with equivalent functions in the clinical environment. The recurrence rate following RFA may also be affected by the hepatologist's skill [32]. However, a study reported no difference between trainees who underwent the carefully monitored training program and mentors in performing RFA [32]. Additionally, we were unable to fully analyze the reasons for the low intrahepatic distant recurrence rate in the linear group. However, our findings remain significant for patients with HCC and hepatologists.

## 5 Conclusion

In conclusion, the linear mode of the RFA protocol results in a low rate of intrahepatic distant recurrence in patients with HCC.

## Ethics Statement

The study was reviewed and approved by guidelines of the Declaration of Helsinki and approved by the Ethics Committee of Nara Medical University (protocol code: 1453; date of approval: January 13, 2017) and Nara Prefecture Seiwa Medical Center (protocol code: 230; date of approval: March 8, 2023).

## Consent

Informed consent was obtained from all patients on an opt‐out basis.

## Conflicts of Interest

The authors declare no conflicts of interest.

## Data Availability

Informed consent for data sharing was not obtained, but the presented data are anonymized, and the risk of identification is low.
